# Nano‐Dual‐Phase Metallic Glass Film Enhances Strength and Ductility of a Gradient Nanograined Magnesium Alloy

**DOI:** 10.1002/advs.202001480

**Published:** 2020-08-16

**Authors:** Chang Liu, Yong Liu, Qing Wang, Xiaowei Liu, Yan Bao, Ge Wu, Jian Lu

**Affiliations:** ^1^ Department of Mechanical Engineering City University of Hong Kong Hong Kong China; ^2^ Max‐Planck‐Institut für Eisenforschung Max‐Planck‐Straße 1 Düsseldorf 40237 Germany; ^3^ Key Laboratory of Near Net Forming of Jiangxi Province Nanchang University Nanchang 330031 P. R. China; ^4^ Laboratory for Microstructures Institute of Materials Science Shanghai University Shanghai 200072 China; ^5^ Institute of Technological Sciences Wuhan University Wuhan 430072 China; ^6^ Hong Kong Branch of National Precious Metals Material Engineering Research Centre City University of Hong Kong Hong Kong China; ^7^ Department of Materials Science and Engineering City University of Hong Kong Hong Kong China; ^8^ Centre for Advanced Structural Materials City University of Hong Kong Shenzhen Research Institute Greater Bay Joint Division Shenyang National Laboratory for Materials Science Shenzhen 518057 China

**Keywords:** gradient nanograined materials, grain growth, magnesium alloys, metallic glasses, shear bands

## Abstract

Magnesium (Mg) alloys are good candidates for applications with requirement of energy saving, taking advantage of their low density. However, the fewer slip systems of the hexagonal‐close‐packed (hcp) structure restrict ductility of Mg alloys. Here, a hybrid nanostructure concept is presented by combining nano‐dual‐phase metallic glass (NDP‐MG) and gradient nanograin structure in Mg alloys to achieve a higher yield strength (230 MPa, 31% improvement compared with the reference base alloy) and larger ductility (20%, threefold higher than the SMAT‐H sample), which breaks the strength–ductility trade‐off dilemma. This hybrid nanostructure is realized by surface mechanical attrition treatment (SMAT) on the surface of a crystalline Mg alloy, and followed by physical vapor deposition of a Mg‐based NDP‐MG. The higher strength is provided by the nanograin layer generated by SMAT. The larger ductility is a synergistic effect of multiple shear bandings and nanocrystallization of the NDP‐MG, inhibition of crack propagation from the SMATed nanograined structure by the NDP‐MG, and strain‐induced grain growth in the SMATed nanograin layer. This hybrid nanostructure design provides a general route to render brittle alloys stronger and ductile, especially in hcp systems.

## Graphical abstract

The work reports a generic solution to an ever‐lasting challenge in structural materials, i.e., overcoming strength–ductility trade‐off. The great leap forward here is realized by the development of a hybrid nanostructure combining the heterogeneous metallic glasses and gradient crystalline grains concepts. This work provides a guideline to render brittle alloys stronger and ductile, especially in hexagonal‐close‐packed (hcp) systems.

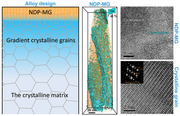

In aerospace and automotive industries, the use of magnesium (Mg) alloys is increasing due to their high strength‐to‐weight ratio.^[^ ^1^ ^]^ However, conventional Mg alloys show poor plastic formability at room temperature, as their hexagonal‐close‐packed (hcp) structure cannot provide enough slip systems to deform plastically.^[^ ^2^ ^]^ The intrinsic mechanism of the low ductility has been revealed by using molecular dynamics simulations^[^ ^3^ ^]^ and experimental investigations^[^ ^4^ ^]^ of microstructure. Plastic deformation of hcp materials is intrinsically governed by dislocation slip and deformation twinning.^[^ ^5^ ^]^ It is suggested that a rich set of the easy‐glide pyramidal 〈c+a〉 dislocations transform to immobile dislocations which serve as obstacles for all other dislocations, limiting *c*‐axis plastic strain. It is also known that the 〈c+a〉 dislocation has higher critical resolved shear stress than certain twinning modes. The deformation twinning is preferred over 〈c+a〉 dislocations in coarse grained Mg alloys,^[^ ^6^ ^]^ thus limiting the ductility. In order to improve mechanical properties, some innovative approaches have been adopted toward microstructure design. For instance, precipitation can effectively increase the strength.^[^ ^7^ ^]^ 〈c+a〉 cross‐slip enhancement by specific dilute solute additions (such as Y, Al, and Zn)^[^ ^8^ ^]^ and the deviation from the hcp to body‐centered‐cubic (bcc) structure (by alloying with Li)^[^ ^9^ ^]^ result in improved ductility. Grain refinement promotes the activation of 〈c+a〉 dislocations compared with deformation twinning,^[^ ^10^ ^]^ thus benefiting the ductility of the fine‐grained Mg alloys. As a method of surface nanocrystallization, surface mechanical attrition treatment (SMAT) is one of the most effective approaches to strengthen materials via introducing a gradient nanograin structure near the treated surface.^[^ ^11^ ^]^ Previous studies have found that microhardness^[^ ^12^ ^]^ and wear behavior^[^ ^13^ ^]^ of Mg alloys can be enhanced by SMAT. However, the introduction of gradient nanograin structure in Mg alloys always compensates the ductility.^[^ ^14^ ^]^ The yield strength of a Mg‐3Gd alloy sharply increases from 70 to 128 MPa after SMAT for only 2 min, but the ductility dramatically decreases to less than 10%.^[^ ^15^ ^]^ The trade‐off of the ductility in Mg alloys is ascribed to the early brittle fracture in the nanograin layer near the surface.^[^ ^14^ ^]^ We hypothesize to inhibit the cracking of nanograin layer to ductilize the gradient nanograined Mg alloys. In the previous research, metallic glass (MG) films were successfully deposited on the surface of brittle materials (such as bulk MGs) to enhance deformability in confined conditions, e.g., bending,^[^ ^16^ ^]^ by triggering multiple shear banding behavior. However, MG films are difficult to enhance the deformability of the matrix alloy in tension.^[^ ^17^ ^]^ Heterogeneous structural MGs or nanoglasses^[^ ^18^ ^]^ are known to possess intrinsic ductility compared with conventional MGs. Inspired from this, we developed a Mg‐based nanodual‐phase metallic glass (NDP‐MG) and deposited it on the top of the gradient nanograined Mg alloy to enhance strength and ductility simultaneously. The high strength is due to introduction of the nanograin layer by SMAT. The NDP‐MG effectively impedes crack propagation from the nanograin layer and undergoes multiple shear‐banding and nanocrystallization, meanwhile, the nanograin layer experiences grain growth and the interior matrix of the Mg alloy provides strain hardening, thus enhancing ductility of the hybrid nanostructural Mg alloy.

A Mg–Zn–Ca NDP‐MG with a thickness of 13 µm was deposited on two side surfaces of the SMATed Mg alloy using magnetron sputtering. Atom probe tomography (APT) investigation shows that the NDP‐MG has an average composition of Mg_57_Zn_36_Ca_7_ (at%). The NDP‐MG exhibits a spiral columnar structure (**Figure** **1**a), in which Ca is enriched in the ≈10 nm thick interface regions (Figure 1b,c). These interface regions divide the Ca‐depleted regions into ≈60 nm diameter substructures. Interestingly, these regions are both amorphous, as indicated by the maze‐like pattern in the high‐resolution transmission electron microscope (HRTEM) image (Figure 1d). The composition of the two regions are Mg_59_Zn_37_Ca_4_ (at%) and Mg_54_Zn_27_Ca_19_ (at%), respectively, both are good glass‐formers.^[^ ^19^, ^20^ ^]^ The brighter contrast of the Mg_54_Zn_27_Ca_19_ interface regions in HRTEM image (Figure 1d) may result from a lower density configuration.^[^ ^21^ ^]^ The heterogeneous nanostructure has a potential to trigger multiple shear banding deformation mechanism,^[^ ^18^ ^]^ and is expected to provide good ductility for the newly developed NDP‐MG.

**Figure 1:**
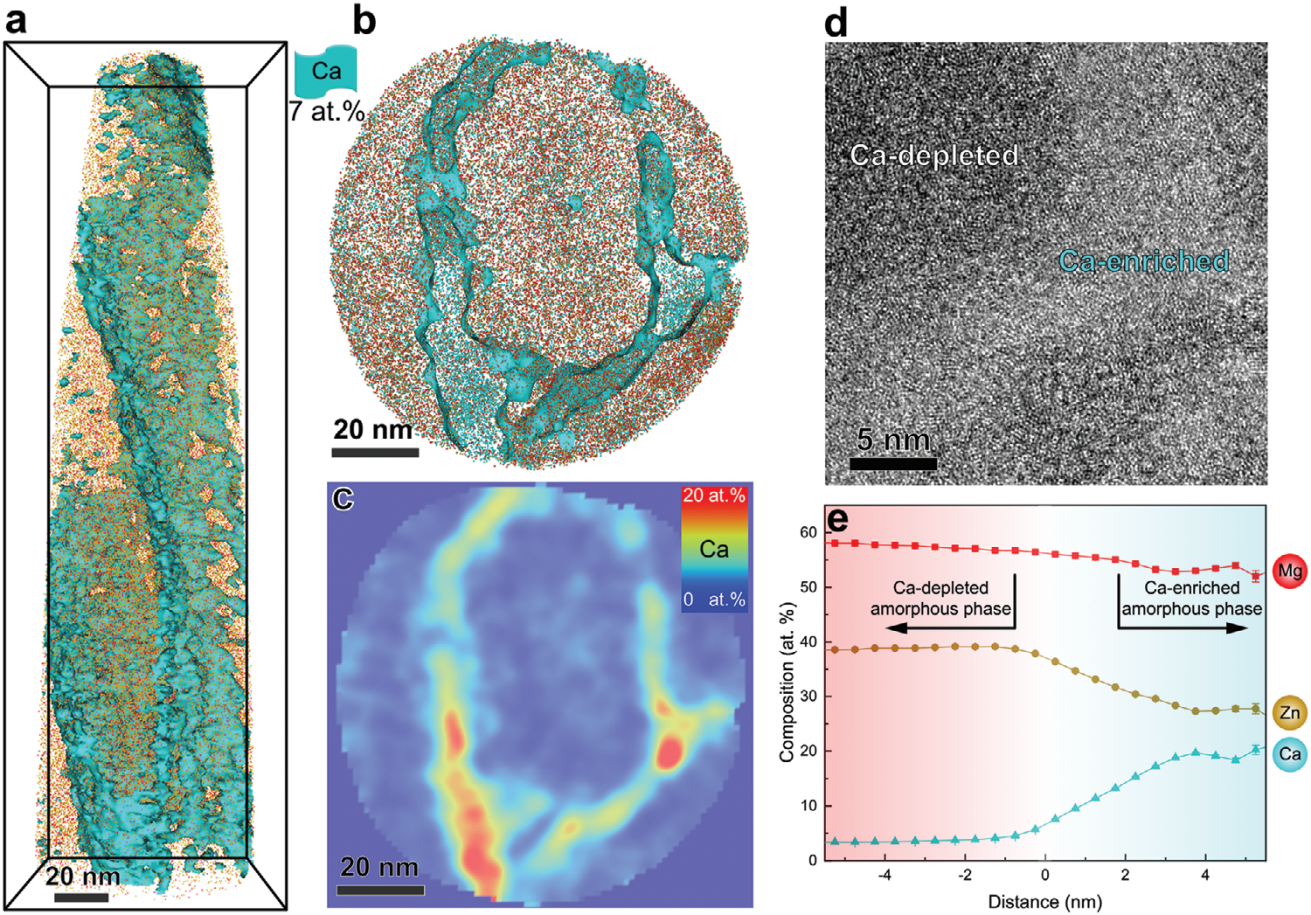
Structure and composition of the Mg‐based NDP‐MG. a) 3D reconstruction of an APT dataset, showing a spiral columnar structure in which Ca is enriched in the interface regions. The Ca‐enriched regions are highlighted by an iso‐composition surface with a 7 at% Ca threshold value. b) A 5‐nm‐thick plane‐view slice from the APT dataset in (a), revealing the Ca‐enriched interface regions. c) The corresponding 2D contour plot of (b) in terms of Ca content. d) Plane‐view HRTEM image shows ≈5 nm thick Ca‐enriched amorphous phase (brighter regions) appearing between Ca‐depleted amorphous regions (darker). e) 1D compositional proxigram generated using 7 at% Ca iso‐composition surface, quantitatively showing compositions of the Ca‐enriched and depleted regions. The error bars represent statistical errors in terms of the standard deviations.

The material beneath the NDP‐MG is a crystalline Mg alloy pretreated by SMAT, comprising a gradient crystalline layer with a thickness of ≈350 µm (**Figure** **2**). Two SMATed Mg alloy were prepared, i.e., SMAT‐L and SMAT‐H with 11 min and 60 min treatment, respectively. The hardness of the top nanograin layer (20 µm depth from surface) is higher (2.2 GPa for SMAT‐H sample and 1.7 GPa for SMAT‐L sample), and gradually decreases toward the interior of the alloy (1.3 GPa). The higher hardness of the top nanograin layer is mainly due to grain refinement.^[^ ^14^, ^22^ ^]^ The high‐energy impact of the SMAT balls reduces the grain size to 100 nm in this Mg alloy system (Figure 2a). Furthermore, the nanograins have weak textures and contain few dislocation (Figure 2a,b), which is promoted by dynamic recrystallization process during SMAT.^[^ ^22^ ^]^

**Figure 2:**
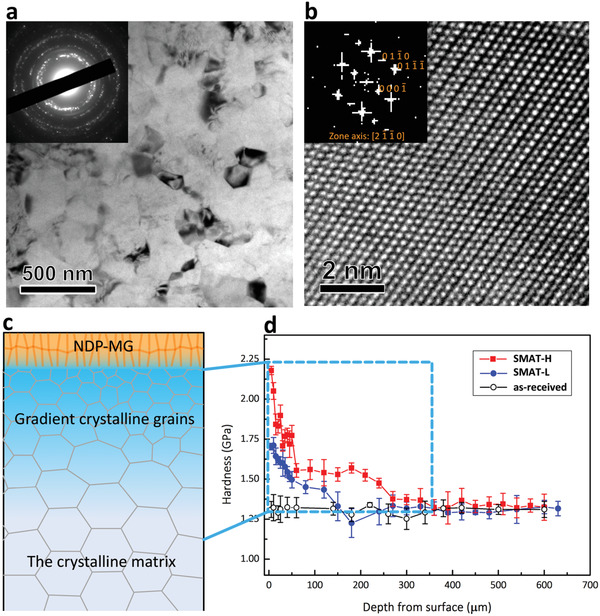
Gradient grain structure of the SMATed Mg alloy. a) Typical bright‐field TEM image of the nanograin layer, probing from 20 µm depth from the surface of the SMAT‐H Mg alloy. The inset shows a selected area electron diffraction (SAED) pattern. The ring feature in the SAED pattern indicates a weak crystallographic texture. b) HRTEM image of a nanograin, probing from [2 ‐1 ‐1 0] zone axis. The inset is the corresponding fast Fourier transform image. c) Schematic illustration of the hybrid nanostructure, showing NDP‐MG on the top of the gradient crystalline grains, followed by the crystalline matrix in the interior. d) Hardness values measured at different depths from the surface of the as‐received, SMAT‐L and SMAT‐H Mg alloys.

The introduction of a gradient nanograin layer increases the yield strength of the Mg alloy in tension (**Figure** **3**). Moreover, higher strength (235 MPa for SMAT‐H sample compared with 175 MPa for as‐received sample) is achieved by applying a longer treatment time in SMAT process, corroborating with a higher hardness of the top nanograin layer (Figure 2d). However, the ductility of the Mg alloy severely decreases after SMAT. The strength–ductility relationships of the base and SMATed Mg alloys show a strength–ductility trade‐off. Fcc alloys, e.g., Cu^[^ ^23^ ^]^ and stainless steels,^[^ ^24^ ^]^ can have increased strength without sacrificing too much ductility via introducing gradient nanograined structure. The enhanced mechanical properties are mainly attributed to grain growth of the nanograin layer^[^ ^23^, ^25^ ^]^ and strain hardening of the interior material^[^ ^25^ ^]^ during plastic deformation. However, Mg alloy, with hcp structure, is difficult to maintain large ductility after introducing gradient nanograined structure, although the strength may increase. It is reported that the ductility of a Mg alloy decreased to less than 10% after SMAT,^[^ ^14^ ^]^ which is similar to the current reference alloys (SMAT‐L and SMAT‐H). Interestingly, if the Mg‐based NDP‐MG film was deposited on the SMAT‐H sample, the ductility dramatically increases to 20%, while maintaining the high yield strength of 230 MPa. The Mg‐based NDP‐MG was also deposited on the surface of an SMAT‐H Cu to reveal the universality of this hybrid nanostructure approach on enhanced strength–ductility synergy (Figure S1, Supporting Information). This indicates that the ductility improvement of the gradient nanograined alloy in the current hybrid nanostructure design is ascribed to the heterogeneous amorphous structure of the NDP‐MG, which will be discussed as below.

**Figure 3:**
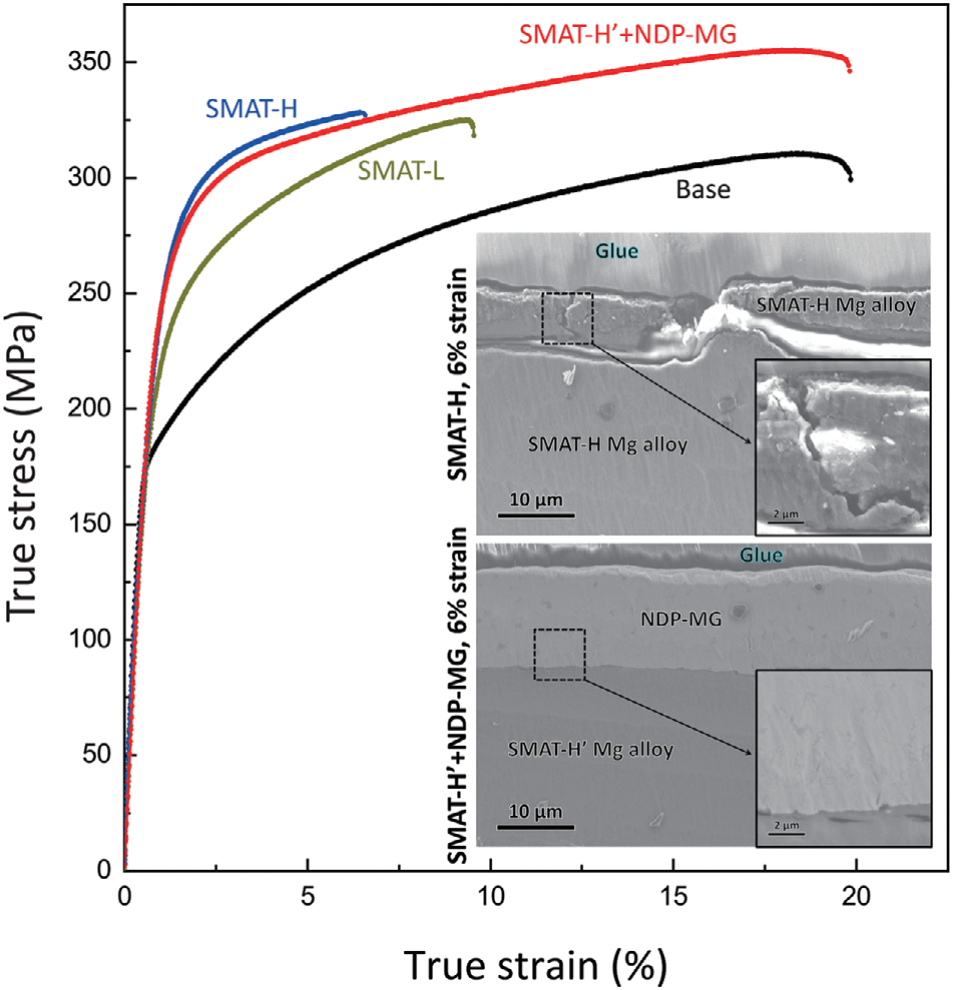
Mechanical properties of the NDP‐MG coated SMAT‐H’ Mg alloy at room temperature. True stress–strain curves of the base (black), SMAT‐L (dark yellow), SMAT‐H (blue), and NDP‐MG coated SMAT‐H′ (red) Mg alloys. The insets are cross‐sectional SEM images of the SMAT‐H and NDP‐MG coated SMAT‐H′ Mg alloys with 6% true strain, showing crack propagation impeding by the NDP‐MG.

After deformation with 6% true strain, large cracks were generated from the surface of the SMAT‐H Mg alloy, and certain parts of the top nanograin layer are even delaminated (inset, Figure 3). These behaviors represent the brittleness of the nanograin layer generated by SMAT. The surface cracks could penetrate into the interior of the alloy and accelerate fracture.^[^ ^26^ ^]^ By contrast, the cracking of the nanograin layer is effectively impeded by the NDP‐MG film (inset, Figure 3). Upon a true strain of 6%, the NDP‐MG film and the nanograin layer on the NDP‐MG coated SMAT‐H′ alloy are still intact with each other, distinct from the surface material delamination on the alloy without NDP‐MG. The conventional MG usually fails at only 2% true strain.^[^ ^27^ ^]^ The current NDP‐MG, however, does not reveal large cracks at a true strain of 6%, which indicates the intrinsic large plastic deformation capacity of the NDP‐MG.

Indeed, plenty of multiple shear bands were generated on the surface of the deformed NDP‐GC (with a true strain of 6%, Figure S2, Supporting Information, and **Figure** **4**b). The shear bands have weaker directionality with respect to the loading direction (Figure S2, Supporting Information) compared with those on the stretched monolithic MG film.^[^ ^17^ ^]^ This implies the deflecting and deferring of shear band propagation via the heterogeneity design. The interface regions of the NDP‐GC are enriched in Ca (Figure 1), and the Ca‐rich MG is known to exhibit a lower strength than that of the Mg‐rich MG in the Mg–Zn–Ca system.^[^ ^20^ ^]^ Therefore, plastic deformation could be easier to take place in the Ca‐enriched regions through shear bands nucleation. The propagation of the shear bands is then hindered by the Ca‐depleted regions, inducing shear band multiplication^[^ ^18^ ^]^ toward different directions. This deformation behavior not only restricts crack formation in the NDP‐GC, but effectively impedes crack generation in the nanograin layer beneath the NDP‐GC as well.

**Figure 4:**
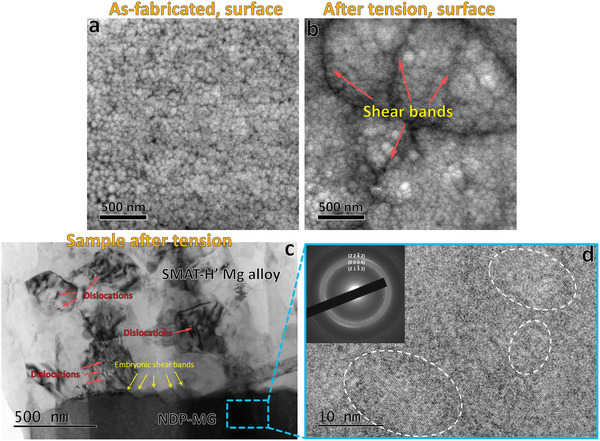
SEM surface morphologies of the NDP‐MG a) before and b) after tension with 6% true strain, showing multiple shear bands generated from the interface regions. c) Cross‐sectional TEM image of the NDP‐MG deposited SMAT‐H′ alloy with 20% true strain, revealing grain growth and dislocation generation in the nanograin layer, and embryonic shear band generation in the NDP‐MG. d) HRTEM image of the deformed NDP‐MG, showing nanocrystallization phenomenon. The inset is the corresponding SAED pattern.

TEM investigations were conducted on the deformed samples to unveil the deformation mechanisms. The grain size of the nanograin layer beneath the NDP‐MG increases from the initial ≈100 to ≈500 nm after deformation (Figure 4c) with 20% true strain. High‐density dislocations are observed in the deformed nanograins, contrast with the quasi‐dislocation free nature of the undeformed nanograins (Figure 2a,b). These phenomena indicate that grain boundary migration and dislocation activities dominate the plastic deformation of the nanograin layer. Conventional nanocrystalline materials possess limited ductility, which is usually due to instability of the grain boundaries^[^ ^28^ ^]^ that induces strain softening. In the current alloy, however, the dislocation motion and pile‐up in the interior matrix^[^ ^25^ ^]^ provide strain hardening, compensating the softening caused by grain growth. Furthermore, the increased grain size is helpful to suppress grain boundary instability, which postpones crack generation and enhances ductility. We note that plenty of embryonic shear bands are generated in the NDP‐MG (Figure 4c). This behavior is distinct from single shear banding of conventional monolithic MGs,^[^ ^27^ ^]^ and prohibits catastrophic failure after yielding. The embryonic shear bands observed in TEM corresponds well with the SEM investigation (Figure 4b). Moreover, strain induced nanocrystallization takes place in the NDP‐MG after deformation (Figure 4d). The nanocrystalline phase is identified to be hcp Ca_2_Mg_6_Zn_3_ by SAED. The NDP‐MG reveals crystalline‐amorphous heterostructure after deformation. The shear band could be initiated from the softer zone of the heterostructure, but its transmission may be delayed by deflecting effect from the harder zone.^[^ ^29^ ^]^ The strain delocalization mechanism of the heterostructure has been proven to improve ductility of the MGs.^[^ ^30^, ^31^ ^]^ Additionally, it is difficult to locate dislocations at the interface region between the NDP‐MG film and the nanograin layer (Figure 4c). This suggests that dislocations could penetrate the nanograin and annihilate at the crystal‐glass interface during deformation. In fact, the amorphous regions in the crystal‐glass composite systems are known to serve as structural sinks for dislocations.^[^ ^31^, ^32^ ^]^ The continuous movement and annihilation of the dislocations in the interfacial region prevents dislocation pile‐up induced strain localization, thus reducing stress peaks and preventing crack generation from the nanograin layer. Meanwhile, the nanograin layer beneath the NDP‐MG experiences grain growth, contributing to large ductility (Figure 4c).

The grain growth in the top nanograin layer is one of the important mechanisms that account for the high ductility of the gradient nanograined fcc alloys.^[^ ^25^ ^]^ It is also important that plastic incompatibility and strain gradient exist at heterointerfaces of the trans‐scale gradient structures upon loading. The geometrically necessary dislocations could be generated near heterointerfaces to comply with plastic deformation, which further interact with the incident dislocations, promoting the heterogeneous deformation‐induced strengthening and hardening.^[^ ^33^ ^]^ The synergistic effects of grain growth on the top and strain hardening in the gradient structure^[^ ^23^ ^]^ contribute to the high strength and large ductility. However, the grain growth phenomenon cannot be realized in the gradient nanograined hcp (such as Mg) alloys, which results in early cracking from the top nanograin layer.^[^ ^14^ ^]^ This behavior severely restricts ductility. The NDP‐MG film coated on the surface has higher ductility than conventional monolithic MGs. Multiple shear bandings postpone the cracking from the NDP‐MG, thus further impeding crack growth from the nanograin layer beneath it. Therefore, the strain delocalization by the NDP‐MG on the surface changes the deformation mechanism of the gradient nanograined hcp Mg alloys from cracking to grain growth at the surface, similar to that of the gradient nanograined fcc alloys. It is worthwhile noting that if one ductile coating has a lower strength, cracking can easily penetrate through it,^[^ ^34^ ^]^ inducing fracture of the entire specimen. Therefore, the coating should have higher strength and good ductility. Furthermore, the adhesion between the coating and the crystalline Mg alloy should be high enough to avoid delamination of the film during deformation. In industry, if the coating has large compositional difference from the substrate, a gradient transition layer with compositions close to both sides of the materials would be introduced to enhance the adhesion,^[^ ^35^ ^]^ taking advantage of stronger chemical bonding between similar atoms. In the current hybrid nanostructured Mg alloy, both the coating and the crystalline alloy are mainly composed of Mg, which facilitates good adhesion between them. In fact, the coating still adheres well on the Mg crystalline alloy after 20% true straining.

Mg alloys can be strengthened by introducing gradient nanograined structure. However, this strategy is unable to provide large ductility in Mg alloys, as restricted by the hcp structure. We developed a hybrid nanostructured Mg alloy via depositing a Mg‐based NDP‐MG film on the surface of the gradient nanograined Mg alloy. The ductility can be dramatically increased to 20%, which is comparable to that of the as‐received base alloy. Meanwhile, the yield strength maintains at 230 MPa (similar to that of the SMAT‐H sample), revealing 31% improvement than the base alloy. The synergistic effects of multiple shear bandings and crystallization of the NDP‐MG, crack impediment by the NDP‐MG, and grain growth of the SMATed nanograin layer promote the large plastic deformation. These mechanisms facilitate excellent mechanical properties in Mg alloys, overcoming the strength–ductility trade‐off dilemma. This hybrid nanostructure alloy design strategy by combining concepts of heterogeneous MG and gradient nanograined structure could be applied to other alloy systems (Figure S1, Supporting Information) to achieve high strength and large ductility.

## Experimental Section

##### Fabrication of the Materials

Mg–3Al–1Zn (wt%, AZ31) alloy sheet with 1.6 mm thickness was selected as base alloy in this study. SMAT was performed by using 3 mm diameter ZrO_2_ balls with a vibration frequency of 20 kHz at room temperature on each side of the alloy sheet. The treatment time for SMAT‐L and SMAT‐H is 11 and 60 min, respectively. After treatment, the thickness of SMAT‐H and SMAT‐L sample become 1.5 and 1.56 mm, respectively. The SMATed Mg alloy sheets were polished with thickness decreased by <2 µm to get rid of pollution and oxide generated in SMAT process. Pure copper sheet (99.99% purity) with a thickness of 1 mm was processed by using SMAT on each side for 5 min. The treatment time is much longer compared with the former report,^[^ ^36^ ^]^ which is selected for largely improving the yield strength but sacrificing the ductility as a compromise. Then Mg_57_Zn_37_Ca_6_ (at%) NDP‐MG with a thickness of 13 µm was deposited on both sides of the SMATed Mg alloy and Cu using magnetron sputtering. In the sputtering process, Ar pressure was 0.5 Pa, substrate bias voltage was −50 V, and the deposition rate was 54 nm min^−1^.

##### Structural and Compositional Characterization

The structures of the Mg‐based NDP‐MG and SMATed Mg alloy were studied by SEM (FEI's Quanta 450 field emission SEM) and TEM (2100F FEG TEM (JEOL), operated at 200 kV). TEM foils with initial thickness of ≈20 µm were prepared from both plane‐view surface and cross‐section of the alloy, and then were ion milled at a temperature of −50 °C to avoid crystallization. Needle‐shaped specimens required for APT were fabricated by lift‐outs and annular milled by focus ion beams. The APT measurements were performed in a local electrode atom probe (CAMEACA LEAP 5000XR). The APT specimens were analyzed at 40 K in laser mode, a pulse repetition rate of 200 kHz, a laser power of 15 pJ, and an evaporation detection rate of 0.3% atom per pulse. Imago Visualization and Analysis Software version 3.8.4 was used for creating the 3D reconstructions and data analysis.

##### Mechanical Characterization

The samples for uniaxial quasi‐static tension test were cut into dog‐bone shape with the gauge dimension of 25 × 6 mm^2^ and then were tested on MTS RT/30 Electro‐Mechanical Material Testing System with a strain rate of 6.8 × 10^−4^ s^−1^ at room temperature. Mg alloys for nanoindentaion were grind and polished to possess mirror‐like surface. Nanoindentation was performed on the samples by using Hysitron's TI950 nanoindenter with a Berkovich tip. Each equivalent position was indented for five times with 1 mN load.

## Conflict of Interest

The authors declare no conflict of interest.

## Supporting information

Supporting Information
