# Supplementary material for: Nano‐Dual‐Phase Metallic Glass Film Enhances Strength and Ductility of a Gradient Nanograined Magnesium Alloy
Source: Adv Sci (Weinh). 2020 Aug 16;7(19):2001480. doi: 10.1002/advs.202001480 (PMC7539178; doi:10.1002/advs.202001480)
Supplement: Supplementary file 1 — Supporting Information [file ADVS-7-2001480-s001.pdf]

© 2020 Wiley-VCH GmbH

ADVANCED SCIENCE
Open Access

Supporting Information

for *Adv. Sci.,* DOI: 10.1002/advs.202001480

Nano-Dual-Phase Metallic Glass Film Enhances Strength and Ductility of a Gradient Nanograined Magnesium Alloy

Chang Liu, Yong Liu, Qing Wang, Xiaowei Liu, Yan Bao, Ge Wu, and Jian Lu**

Copyright WILEY-VCH Verlag GmbH & Co. KGaA, 69469 Weinheim, Germany, 2020.

Supporting Information

Nano-dual-phase metallic glass film enhances strength and ductility of a gradient nanograined Magnesium alloy

Chang Liu, Yong Liu, Qing Wang, Xiaowei Liu, Yan Bao, Ge Wu, Jian Lu**

Dr. C. Liu, Prof. Y. Liu, Prof. Q. Wang, Dr. X. Liu, Mr. Y. Bao, Dr. G. Wu, Prof. J. Lu
Department of Mechanical Engineering, City University of Hong Kong, Hong Kong, China
Email: ge.wu@mpie.de (G. Wu); jianlu@cityu.edu.hk (J. Lu)

Dr. C. Liu, Dr. G. Wu
Max-Planck-Institut für Eisenforschung, Max-Planck-Straße 1, 40237 Düsseldorf, Germany

Prof. Y. Liu
Key Laboratory of Near Net Forming of Jiangxi Province, Nanchang University, Nanchang 330031, PR China

Prof. Q. Wang
Laboratory for Microstructures, Institute of Materials Science, Shanghai University, Shanghai 200072, China

Dr. X. Liu
Institute of Technological Sciences, Wuhan University, Wuhan 430072, China

Prof. J. Lu
Hong Kong Branch of National Precious Metals Material Engineering Research Centre, City University of Hong Kong, Hong Kong, China
Department of Materials Science and Engineering, City University of Hong Kong, Hong Kong, China
Centre for Advanced Structural Materials, City University of Hong Kong Shenzhen Research Institute, Greater Bay Joint Division, Shenyang National Laboratory for Materials Science, Shenzhen 518057, China

Mechanical property of the NDP-MG coated SMAT Cu

The Mg-based NDP-MG was also deposited on the surface of a SMAT-H Cu to reveal the universality of this hybrid nanostructure approach on enhanced strength-ductility synergy. In order to emphasize the ductilizing effect of the NDP-MG, we used a vigorous SMAT treatment on Cu, which is different from the previous report that the yield strength of Cu has certain improvement with maintained ductility by using moderate SMAT[1] or surface mechanical grinding treatment (SMGT).[2] This vigorous SMAT treatment dramatically increases the yield strength (from 48 MPa to 235 MPa) of Cu but severely reduces the ductility (from 42% to 19%). The deposition of Mg-based NDP-MG film on the SMAT-H Cu increases the ductility to 29%, while maintaining the high yield strength of 230 MPa (Figure S1). The ductility of the NDP-MG coated SMAT-H' Cu does not reach the level of the as-prepared Cu, which may be due to the weaker chemical bonding between Cu and Mg-based NDP-MG. It is worthwhile noting that a Ni-P monolithic MG film has been deposited on the surface of a gradient nanograined Ni pre-treated by using SMGT.[3] However, the tensile ductility of the Ni-P MG coated SMGT Ni reveals notable reduction compared with that of the SMGT Ni. This indicates that the ductility improvement of the gradient nanograined alloy in the current hybrid nanostructure design is ascribed to the heterogeneous amorphous structure of the NDP-MG.

References

[1] X. Liu, K. Wu, G. Wu, Y. Gao, L. Zhu, Y. Lu, J. Lu, Scripta Mater. 2016, 124, 103.

[2] T. Fang, W. Li, N. Tao, K. Lu, Science 2011, 331, 1587.

[3] X. Lu, Q. Lu, Y. Li, L. Lu, Sci. Rept. 2013, 3, 3319.

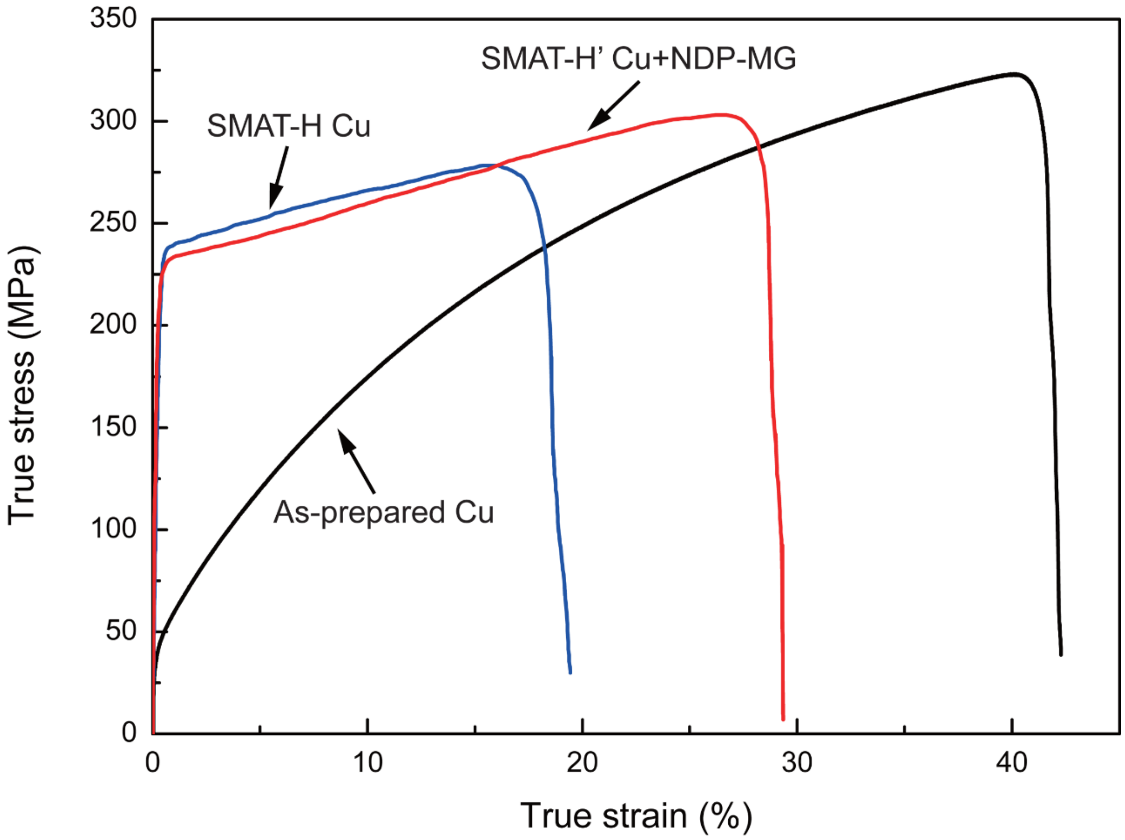

Figure S1. True stress–strain curves of the as-prepared Cu (black), SMAT-H Cu (blue) and NDP-MG coated SMAT-H’ Cu (red).

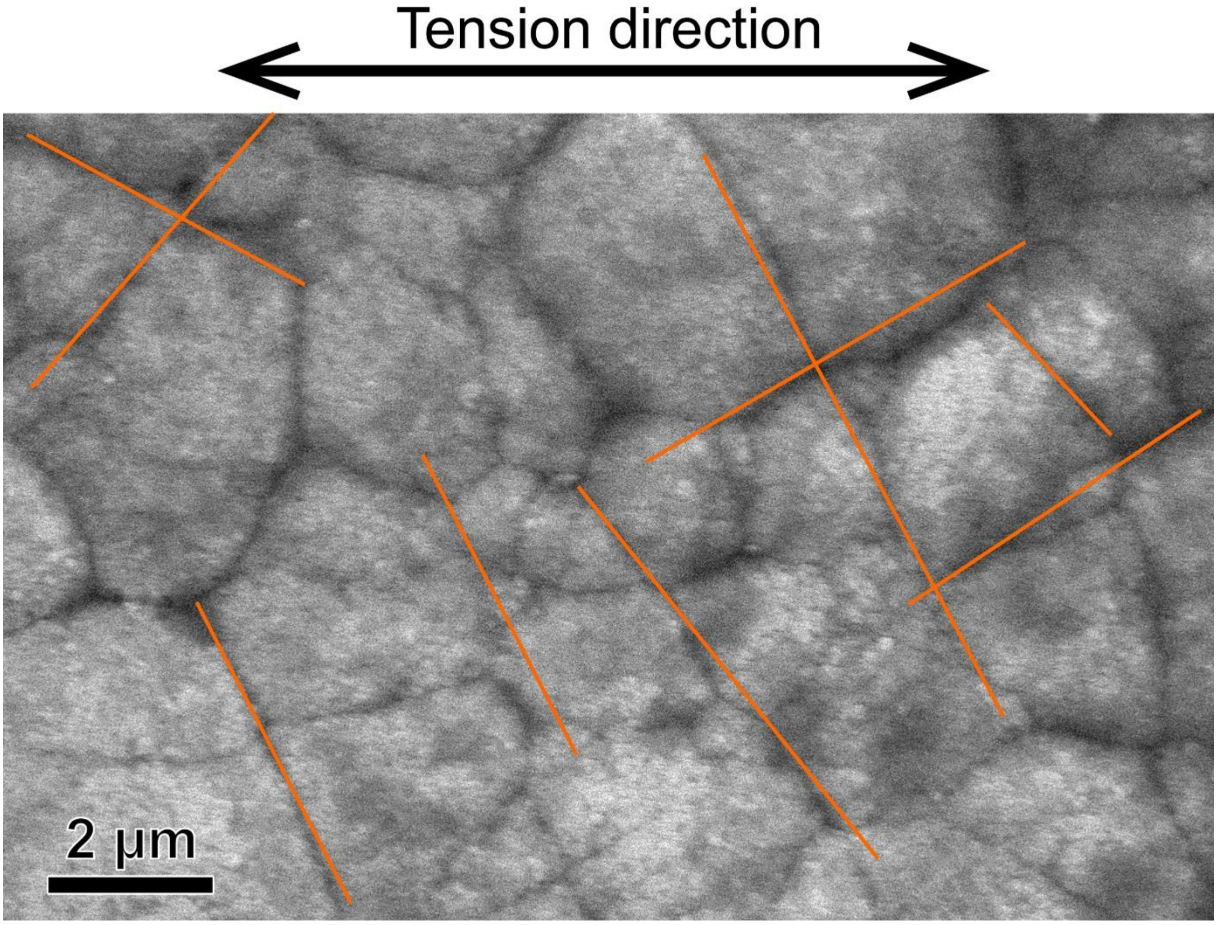

Figure S2. SEM surface morphology of the NDP-MG after tension with 6% true strain. The orange lines mark some of the shear bands.
